# Does a Nurse-Led Program of Support and Lifestyle Management for patients with coronary artery disease significantly improve psychological outcomes among the patients? A meta-analysis

**DOI:** 10.1097/MD.0000000000012171

**Published:** 2018-08-21

**Authors:** Zu-Chun Luo, Lu Zhai, Xia Dai

**Affiliations:** a Department of Internal Medicine Education; b Department of Endocrinology, the First Affiliated Hospital of Guangxi Medical University, Nanning, Guangxi, China.

**Keywords:** anxiety, coronary heart disease, depression, nurse-coordinated prevention program, nurse-led patient management

## Abstract

**Background:**

Nowadays, secondary prevention of coronary heart disease (CHD) is commonly provided by nurse-coordinated prevention programs (NCPPs). NCPPs were recommended to be incorporated into the healthcare systems by the European Society of Cardiology (ESC) as stated in their 2012 European Guideline. Even if Nurse-Led Programs of Support and Lifestyle Management are beneficial to the patients with CHD, it is not clear whether these programs significantly improve psychological outcomes among the patients. Therefore, in this analysis, we aimed to systematically compare anxiety and depression reported among CHD patients who were assigned to a Nurse-Led Programs of Support and Lifestyle Management versus patients who were assigned to a normal usual care setting.

**Methods:**

Online databases were searched for English publications assessing anxiety and depression in CHD patients who were assigned to a Nurse Interventional program versus patients who were assigned to a normal usual care setting. This analysis was carried out by RevMan software (version 5.3). For dichotomous data, odds ratios (ORs) and 95% confidence intervals (CIs) were generated whereas for continuous data, weight mean difference (WMDs) and 95% CIs were calculated.

**Results:**

A total number of 3110 patients were analyzed (1526 participants were assigned to the Nurse Interventional group whereas 1584 participants were assigned to the normal usual care group). Patients’ enrollment time period varied from the year 2008 to the year 2015. Results of this analysis showed that depression among participants who were assigned to a Nurse-Led Program of Support and Lifestyle Management was not significantly different (OR: 0.90, 95% CI: 0.68–1.20; *P* = .47) compared to participants who were assigned to the normal usual care setting. When continuous data were used, still no significant difference was observed (WMD: −0.83, 95% CI: −1.68–0.02; *P* = .06). A similar result was obtained even when anxiety was assessed (WMD: −1.38, 95% CI: −3.21–0.45; *P* = .14).

**Conclusions:**

The current analysis did not show any significant improvement in reduction of depression and anxiety among CHD patients who were assigned to a Nurse-Led Program of Support and Lifestyle Management versus those patients who were assigned to a normal usual care setting. Therefore, according to this analysis, even if a Nurse-Led Program of Support and Lifestyle Management might be clinically effective, it does not improve mental well-being in these patients with CHD.

## 1 Introduction

Recently published Epidemiological reports showed an increase in the total number of patients suffering from coronary heart disease (CHD) around the world.^[1]^ Counseling and other primary preventive measures have long been taken to reduce the risk of this chronic disease. For those patients who seek primary medical advice, health tips and benefits in terms of a healthy diet including low salt and low fat diets, regular exercises, smoking cessation, weight control, alcohol consumption and other health counseling related to the reduction of cardiovascular risk factors are advised by healthcare providers. And for those patients who have already been affected, secondary measures have been introduced to control or reduce other risk factors which might aggravate their conditions.^[2]^

Nowadays, secondary prevention of CHD is commonly provided by nurse-coordinated preventive programs (NCPPs).^[3]^ Previous studies have already shown NCPPs to be highly effective in reducing cardiovascular disease risks compared to the usual care and hence, NCPPs were therefore recommended to be incorporated into the healthcare systems by the European Society of Cardiology (ESC) as stated in their 2012 European Guideline.

Nevertheless, patients with CHD are often psychologically affected by their health conditions.^[4]^ Accepting the fact that the heart is sick or is not functioning normally, and being aware that a heart attack might be precipitated at any time could be stressful and thus lead to anxiety and depression among the patients. The patients also develop psychological stress related to the etiology of their disease, development, duration, outcome as well as the prognosis of their current state. Several studies have shown depression to be a risk factor for morbidity and mortality in these patients with CHD, especially after an acute coronary syndrome.^[5]^ Depression is also believed to increase the number of adverse cardiovascular events, the total number of readmission to the hospital, and also increases cardiovascular death. Anxiety is also, to a lesser extent, a contributor to the adverse cardiac events. A recent survey dealing with physical and psychological symptoms showed anxiety to mainly be related with palpitation and arrhythmia which could further aggravate CHD.^[5]^ Therefore, reducing psychological stress among the CHD patients could somehow show some benefits.

Even if Nurse-Led Programs of Support and Lifestyle Management are beneficial to patients with CHD, it is not clear whether these programs significantly improved psychological outcomes among the patients. Therefore, in this analysis, we aimed to systematically compare anxiety and depression reported among CHD patients who were assigned to Nurse-Led Programs of Support and Lifestyle Management versus patients who were assigned to normal usual care settings.

## 2 Methods

### 2.1 Searched databases and strategies

Online (electronic) databases [MEDLINE, EMBASE (www.sciencedirect.com), SCOPUS, PsycINFO, Cochrane Library, and www.ClinicalTrials.gov] were searched for English publications assessing anxiety and depression in CHD patients who were assigned to a Nurse Interventional program versus patients who were assigned to a normal usual care setting after having been discharged from the hospital or during follow-up sessions in person or by phone calls.

The following searched terms were used:

- Nurse care, coronary heart disease;
- Nurse care, coronary heart disease and depression;
- Nurse care, coronary heart disease and anxiety;
- Nurse care, coronary heart disease and psychological outcomes;
- Nurse care, coronary heart disease and outcomes.

The word “coronary heart disease” was interchangeable with the words “coronary artery disease,” “acute coronary syndrome,” “cardiovascular disease” whereas the word “nurse care” was interchangeable with the words “nurse intervention,” “nurse-led programs,” “nurse-assisted programs.”

This search was restricted to articles which were published in and after the year 2000. Articles which were published before the year 2000 were excluded from this analysis. With the drastic development in medicine and technology, we believe that at least treatment, guideline and better quality of clinical services were obtained during and after the year 2000; Also, in meta-analyses, it is better to include data within the recent 10 to 15 years. Therefore, this current analysis was based on studies which were published during and after the year 2000.

### 2.2 Inclusion and exclusion criteria

Inclusion criteria were:

(1) Articles assessing anxiety and depression in CHD patients who were assigned to a Nurse Interventional program versus patients who were assigned to a normal usual care setting;
(2) Articles which were published during and after the year 2000;
(3) Articles reporting data either in the continuous or dichotomous forms.

Exclusion criteria were:

(1) Relevant articles which were published before the year 2000;
(2) Articles which did not report psychological outcomes (depression or anxiety) as their clinical endpoints;
(3) Review articles, case studies, and letters to editors;
(4) Repeated studies.

### 2.3 Types of participants, definitions, outcomes, and follow-ups

All the participants who were involved in this analysis were candidates of CHD.

Nurse Intervention group: consisted of participants who received coaching support intervention or group education or home visit by qualified nurses and individual management plans for CHD and they were repeatedly advised appropriately through phone calls or personal home visits.

Usual Care group: consisted of routine care which was provided by the community health service including brochures containing information concerning the prevention of CHD and other health related information provided at the community health service or care which was provided each time during the follow-up visit in the outpatient department.

The main outcomes of this analysis were:

(1) Depression reported among the CHD patients;
(2) Anxiety reported among the CHD patients.

The follow-up time periods varied in each study. The types of participants, outcomes which were reported and follow-up time periods in each study are listed in Table 1.

**Table 1:**
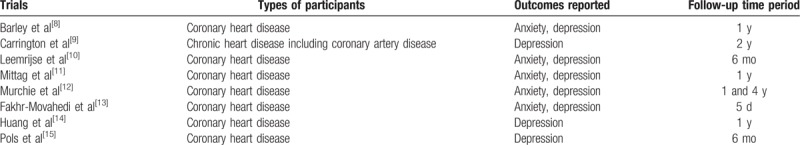
Types of participants, outcomes, and follow-up time periods.

### 2.4 Data extraction and quality assessment

Relevant data were extracted by 3 independent reviewers and included: the type of study, the total number of participants who were assigned to the Nurse Intervention group or the Usual Care group, the types of participants, the time period of patients’ enrollment, the total number of events reporting depression and anxiety, the type of data, the mean and standard deviation reported, and the baseline features of the participants.

The methodological quality of the trials were assessed with reference to the criteria suggested by the Cochrane Collaboration^[6]^ whereby grades were given (grade A represented low risk of bias, grade B represented moderate risk whereas high risk of bias was represented by a grade C).

Any disagreement which occurred during the data extraction process or the methodological assessment was resolved by consensus.

### 2.5 Statistical analysis

This analysis was carried out by the updated version of the RevMan software (version 5.3).

For dichotomous data, that is quantitative data, odds ratios (ORs) and 95% confidence intervals (CIs) were generated whereas for continuous data where mean and standard deviation were provided, weight mean difference (WMDs) and 95% CIs were generated.

Heterogeneity was assessed by the *Q* statistic test whereby a *P*-value less than .05 was considered statistically significant; the *I*^2^ statistic test whereby a higher value of *I*^2^ denoted a high level of heterogeneity (random effects model was used) and a lower value of *I*^2^ denoted a low level of heterogeneity (fixed effects model was used).

Sensitivity analysis was carried out by a method of exclusion whereby each trial was excluded one by one and the new result was compared each time with the actual result to observe any significant difference observed among the results.

### 2.6 Ethical approval

This is a meta-analysis of previously published studies and therefore, ethical approval or any board review approval was not required.

## 3 Results

### 3.1 Searched outcomes

The PRISMA reporting guideline was followed.^[7]^ Following a careful search through the online databases, a total number of 412 publications were obtained. After carefully assessing the titles and abstracts, an initial screening was carried out whereby 369 articles were eliminated.

Forty-three full text articles were assessed for eligibility. Further eliminations were carried out whereby publications were eliminated due to the following reasons:

(1) They were review of the literature (4);
(2) They were case studies (2);
(3) They were articles which were published before the year 2000 (5);
(4) They did not report anxiety and depression (7);
(5) They were duplicated studies (17).

**Figure 1:**
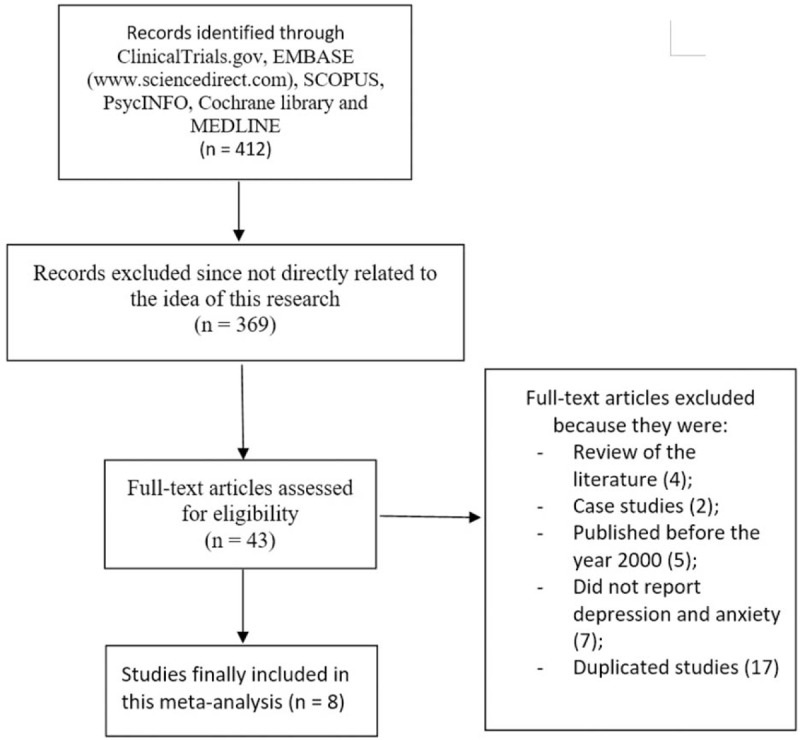
Flow diagram representing the study selection.

Finally, 8 studies^[8–15]^ were finalized for this meta-analysis as shown in Fig. 1.

### 3.2 General and baseline features of the trials

The general and the baseline features of the participants are reported in Tables 2 and 3, respectively.

**Table 2:**
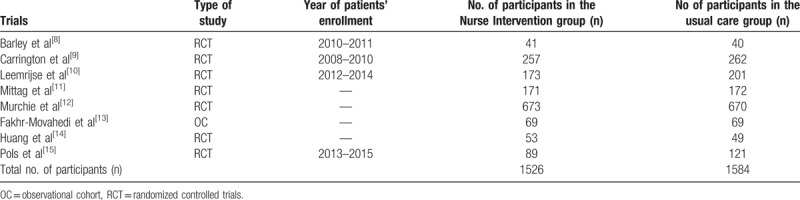
General features of the trials.

**Table 3:**
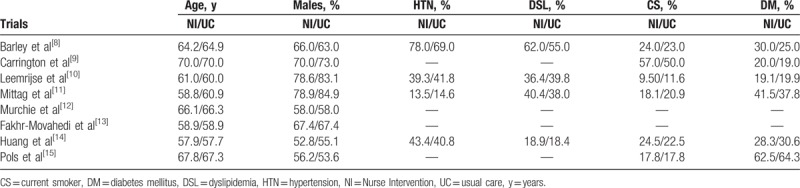
Baseline features of the participants.

Seven trials and one observational cohort were included in this meta-analysis.

A total number of 3110 patients were analyzed (1526 participants were assigned to the Nurse Interventional group whereas 1584 participants were assigned to the normal usual care group).

Patients’ enrollment time period varied from the year 2008 to the year 2015.

The mean age of the participants varied from 57.7 to 70 years. Male participants were predominant. Other cardiovascular risk factors including hypertension, dyslipidemia, current smoker, and diabetes mellitus are reported in Table 3.

According to the baseline features of the participants, there was no significant difference between those who were assigned to the Nurse Intervention group versus the normal Usual Care group.

### 3.3 Main results of this analysis

**Figure 2:**
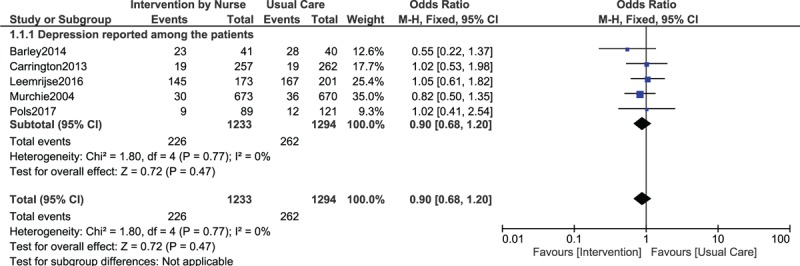
Depression reported in CHD patients who were assigned to a Nurse-Led Program of Support and Lifestyle Management versus those who were assigned to normal usual care (using dichotomous data).

Results of this analysis showed that depression which was reported among participants who were assigned to a Nurse-Led Program of Support and Lifestyle Management was not significantly different (OR: 0.90, 95% CI: 0.68–1.20; *P* = .47) compared to participants who were assigned to the normal usual care setting as shown in Fig. 2 (dichotomous data).

**Figure 3:**
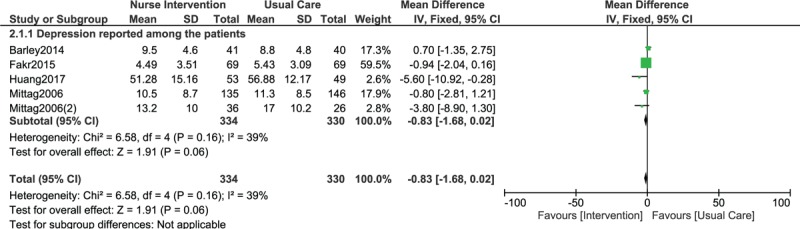
Depression reported in CHD patients who were assigned to a Nurse-Led Program of Support and Lifestyle Management versus those who were assigned to normal usual care (using continuous data).

**Figure 4:**
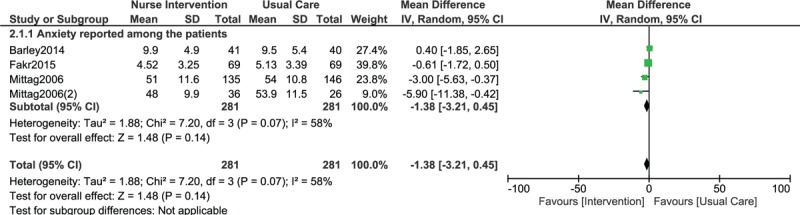
Anxiety reported in CHD patients who were assigned to a Nurse-Led Program of Support and Lifestyle Management versus those who were assigned to normal usual care (using continuous data).

When continuous data was used, still no significant difference was observed among participants who were and who were not assigned to a Nurse-Led Program of Support and Lifestyle Management (WMD: −0.83, 95% CI: −1.68–0.02; *P* = .06) as shown in Fig. 3. A similar result was also obtained when anxiety was assessed (WMD: −1.38, 95% CI: −3.21–0.45; *P* = .14) as shown in Fig. 4.

Sensitivity analysis resulted in consistent results throughout.

## 4 Discussion

Several studies have shown nurse-delivered risk factor intervention programs for patients with CHD to be more effective than the usual care program in terms of lifestyle modifications that might improve cardiovascular risk factors.^[16]^ However, their influence on psychological outcomes has seldom been systematically analyzed.

In this analysis, we aimed to show whether Nurse-Led Programs of Support and Lifestyle Management significantly improved psychological outcomes among patients with CHD.

Results of this analysis did not show any significant difference when depression and anxiety were assessed among patients who were assigned to a Nurse-Led Programs of Support and Lifestyle Management versus patients who were assigned to a normal usual care setting.

Similar to the results of this current analysis, the Coaching patients On Achieving Cardiovascular Health (COACH) trial,^[17]^ which was a multicenter randomized trial in patients with CHD, did not show any impact on depression among the participants. Another integrated community-based nurse-led cardiovascular disease prevention program that included risk factors and lifestyle management, education sessions, exercise and prescription of medications despite showing a significant reduction in depression levels, was not maintained after 1 year.^[18]^ But as recently stated in a comprehensive search (1980–2014), it should be noted that chronic stress and major depression have been shown to be involved with changes in the brain structures (for example, loss of dendritic spines, decreased dendritic arborization, reduced number of glial cells in the hippocampus).^[19]^

While the ALTRA trial which will examine the efficacy of advanced practice nurse-led telehealth rehabilitative program is still under study,^[20]^ another trial further supported the results of this analysis.^[21]^ The study, which was based on patients reported outcomes (the Whole Systems Demonstrator telehealth questionnaire study) which was nested in a pragmatic, cluster randomized trial of telehealth (the Whole Systems Demonstrator telehealth trial) whereby specialized nurses were involved, and where telehealth was compared with usual care, the former did not improve psychological outcomes for the patients with cardiac issues over a follow-up period of 1 year.

Although it is good to know that Nurse-Led Program of Support and Lifestyle Management do not increase depression or anxiety in patients with CHD, psychological supports which are provided by these trained nurses also do not improve mental well-being.

However, other previous studies also showed results that were different from this analysis. In a study whereby a demographic information questionnaire, Spielberger's Two-part Anxiety Scale (STAI) and Beck's Depression Inventory (BDI) were used to collect data, cardiac rehabilitation attained sufficient efficacy to reduce depression 2 months after coronary related cardiac surgery.^[22]^

Results from the RESPONSE randomized controlled trial also showed the involvement of a Nurse Coordinated Prevention Program (NCPP) to have decreased depressive symptoms among patients with acute coronary syndromes, which could further contribute to the well-being and reduction of overall risk of recurrent events.^[23]^

Moreover, in a randomized controlled trial of in-hospital nursing support for first time myocardial infarction patients and their partners showed findings which strongly suggested that a simple counseling program carried out by coronary care nurse in the hospital significantly decreased the rate of anxiety and depression in patients with myocardial infarction as well as for their partners.^[24]^ Nevertheless, the current analysis was different due to the fact the psychological outcomes were assessed not during an in-patient follow-up, but as out-patient, during a longer follow-up time period. In addition, many patients in the current analysis suffered stable CHD, which did not reach a stage of myocardial infarction in majority of cases.

At last, it should not be ignored that the presence of severe anxiety-depressive disorders before cardiac rehabilitation might have an impact on the outcomes.^[25]^

Similar to other studies, this analysis also has limitations. First of all, due to the limited number of participants, the results might have been affected. Secondly, the follow-up time periods were different in different studies. This might also have had an impact on the results which were obtained. It might be possible that a significantly different result be obtained if a long term follow-up time period was considered. Unfortunately the studies which were included in this analysis were not continually followed during the long term, and therefore, we could not carry out an analysis based on a long-term follow-up time period. In addition, publication bias could not be assessed due to the very limited number of studies which were involved in assessing the outcomes. Other generalized limitations would be the fact that not many researches had been carried out based on a Nurse-Led Program of Support and Lifestyle Management as compared to the normal usual care settings. Therefore, restrictions were observed in terms of the total number of participants, the outcomes which were reported, and the total number of studies that were included. Also, we only included studies which were published during and after the year 2000. This might have affected the results. However, previously published studies were not included as stated in the inclusion and exclusion criteria since the caring facilities, and the treatment modalities before and today are not similar. In addition, our meta-analysis was based on recent studies which were published within approximately the recent 15 years.

Also, different categories of patients were included if we look at the different papers that were included in this analysis. In the original study by Barley et al, only CHD patients with actual chest pain and depression were included. This might be another limitation. In general, other limitations which were encountered were the fact that when several of the participants were contacted, a lack of response was observed whether by direct contact or through email and it has been thought that the expected guideline care was not delivered in several cases. Moreover, the behavior and approach of the nurses might have been different with different individuals and a few nurses might have had difficulty applying behavior techniques. In the study published by Carrington et al, the authors specified their inability to standardize clinical profiling data, and some possible confounding factors of differential cardiac interventions as well as the cardiac drugs which were used indicating another possible limitation and that the results would have to be interpreted with caution.

Otherwise, this is a well-conducted meta-analysis which might represent an interesting piece of information to the Medical and Nursing Departments.

## 5 Conclusions

The current analysis did not show any significant improvement in reduction of depression and anxiety among CHD patients who were assigned to a Nurse-Led Program of Support and Lifestyle Management versus those patients who were assigned to a normal usual care setting. Therefore, according to this analysis, even if a Nurse-Led Program of Support and Lifestyle Management might be clinically effective, it does not improve mental well-being in these patients with CHD.

## Author contributions

Zu-chun Luo, Lu Zhai, and Xia Dai were responsible for the conception and design, acquisition of data, analysis and interpretation of data, drafting the initial manuscript and revising it critically for important intellectual content. Zu-chun Luo wrote the final manuscript.

Zu-chun Luo is the first author.

**Conceptualization:** Zu-chun Luo, Lu Zhai, Xia Dai.

**Data curation:** Zu-chun Luo, Lu Zhai, Xia Dai.

**Formal analysis:** Zu-chun Luo, Lu Zhai, Xia Dai.

**Funding acquisition:** Zu-chun Luo, Lu Zhai, Xia Dai.

**Investigation:** Zu-chun Luo, Lu Zhai, Xia Dai.

**Methodology:** Zu-chun Luo, Lu Zhai, Xia Dai.

**Project administration:** Zu-chun Luo, Lu Zhai, Xia Dai.

**Resources:** Zu-chun Luo, Lu Zhai, Xia Dai.

**Software:** Zu-chun Luo, Lu Zhai, Xia Dai.

**Supervision:** Zu-chun Luo, Lu Zhai, Xia Dai.

**Validation:** Zu-chun Luo, Lu Zhai, Xia Dai.

**Visualization:** Zu-chun Luo, Lu Zhai, Xia Dai.

**Writing – original draft:** Zu-chun Luo, Lu Zhai, Xia Dai.

**Writing – review & editing:** Zu-chun Luo, Lu Zhai, Xia Dai.
